# Role of selective V_2_-receptor-antagonism in septic shock: a randomized, controlled, experimental study

**DOI:** 10.1186/cc9320

**Published:** 2010-11-05

**Authors:** Sebastian Rehberg, Christian Ertmer, Matthias Lange, Andrea Morelli, Elbert Whorton, Martin Dünser, Anne-Katrin Strohhäcker, Erik Lipke, Tim G Kampmeier, Hugo Van Aken, Daniel L Traber, Martin Westphal

**Affiliations:** 1 Department of Anesthesiology and Intensive Care, University of Muenster, Albert-Schweitzer-Str. 33, Muenster 48149, Germany; 2 Department of Anesthesiology and Intensive Care, University of Rome, 'La Sapienza', Viale del Policlinico 155, 00161 Rome, Italy; 3 Department of Biostatistics and Epidemiology, University of Texas Medical Branch, 301 University Boulevard, Galveston, TX 77550, USA; 4 Department of Intensive Care Medicine, Inselspital, Medical University of Bern, CH-3010 Bern, Switzerland; 5 Investigational Intensive Care Unit, Department of Anesthesiology, University of Texas Medical Branch, 301 University Boulevard, Galveston, TX 77550, USA

## Abstract

**Introduction:**

V_2_-receptor (V_2_R) stimulation potentially aggravates sepsis-induced vasodilation, fluid accumulation and microvascular thrombosis. Therefore, the present study was performed to determine the effects of a first-line therapy with the selective V_2_R-antagonist (Propionyl_1_-D-Tyr(Et)_2_-Val_4_-Abu_6_-Arg_8,9_)-Vasopressin on cardiopulmonary hemodynamics and organ function vs. the mixed V_1a_R/V_2_R-agonist arginine vasopressin (AVP) or placebo in an established ovine model of septic shock.

**Methods:**

After the onset of septic shock, chronically instrumented sheep were randomly assigned to receive first-line treatment with the selective V_2_R-antagonist (1 μg/kg per hour), AVP (0.05 μg/kg per hour), or normal saline (placebo, each *n *= 7). In all groups, open-label norepinephrine was additionally titrated up to 1 μg/kg per minute to maintain mean arterial pressure at 70 ± 5 mmHg, if necessary.

**Results:**

Compared to AVP- and placebo-treated animals, the selective V_2_R-antagonist stabilized cardiopulmonary hemodynamics (mean arterial and pulmonary artery pressure, cardiac index) as effectively and increased intravascular volume as suggested by higher cardiac filling pressures. Furthermore, left ventricular stroke work index was higher in the V_2_R-antagonist group than in the AVP group. Notably, metabolic (pH, base excess, lactate concentrations), liver (transaminases, bilirubin) and renal (creatinine and blood urea nitrogen plasma levels, urinary output, creatinine clearance) dysfunctions were attenuated by the V_2_R-antagonist when compared with AVP and placebo. The onset of septic shock was associated with an increase in AVP plasma levels as compared to baseline in all groups. Whereas AVP plasma levels remained constant in the placebo group, infusion of AVP increased AVP plasma levels up to 149 ± 21 pg/mL. Notably, treatment with the selective V_2_R-antagonist led to a significant decrease of AVP plasma levels as compared to shock time (*P *< 0.001) and to both other groups (*P *< 0.05 vs. placebo; *P *< 0.001 vs. AVP). Immunohistochemical analyses of lung tissue revealed higher hemeoxygenase-1 (vs. placebo) and lower 3-nitrotyrosine concentrations (vs. AVP) in the V_2_R-antagonist group. In addition, the selective V_2_R-antagonist slightly prolonged survival (14 ± 1 hour) when compared to AVP (11 ± 1 hour, *P *= 0.007) and placebo (11 ± 1 hour, *P *= 0.025).

**Conclusions:**

Selective V_2_R-antagonism may represent an innovative therapeutic approach to attenuate multiple organ dysfunction in early septic shock.

## Introduction

Arginine vasopressin (AVP) is recommended by the Surviving Sepsis Campaign to 'be subsequently added to norepinephrine' in volume- and catecholamine-refractory septic shock [1]. In the randomized, controlled, multicenter Vasopressin and Septic Shock Trial (VASST), however, AVP failed to reduce overall mortality as compared with norepinephrine among patients with septic shock [2].

AVP represents a mixed V_1a_/V_2 _receptor (V_1a_R/V_2_R) agonist with a selectivity of 1:1 for each of these receptors. Whereas particular attention has been paid to the vasoconstriction mediated by vascular V_1a_Rs [3,4], there is increasing evidence that stimulation of extrarenal (endothelial) V_2_Rs [5-7] may aggravate sepsis-induced vasodilation [4,8], fluid accumulation [9], leukocyte rolling [10], and microvascular thrombosis [11]. Against this background, selective V_2_R-antagonism potentially represents a new therapeutic approach in septic shock.

We hypothesized that a first-line therapy with the selective V_2_R-antagonist (propionyl_1_-D-Tyr(Et)_2_-Val_4_-Abu_6_-Arg_8,9_) vasopressin [12,13] is more effective than infusion of placebo and AVP in restoring cardiovascular and renal functions in early ovine septic shock. Open-label norepinephrine was additionally titrated to maintain mean arterial pressure (MAP) in each group if necessary. Therefore, the present study was designed as a prospective, randomized, controlled, laboratory experiment to elucidate the effects of these treatment strategies on cardiopulmonary hemodynamics, mesenteric blood flow, global oxygen transport, acid-base balance, organ function, AVP plasma levels, oxidative stress, and mortality. The study hypothesis was tested in an established ovine model of fulminant septic shock resulting from generalized fecal peritonitis [14,15].

## Materials and methods

### Instrumentation and surgical procedures

After approval by the Local Animal Research Committee, 21 female sheep were anesthetized, mechanically ventilated, and instrumented for chronic hemodynamic monitoring using an established protocol [14,15]. Details on the instrumentation and surgical procedures are provided in the supplemental digital content in Additional file 1.

### Experimental protocol

Following baseline (BL) measurements, autologous feces were injected into the peritoneal cavity via an intraperitoneal suction catheter. When septic shock had developed (so-called 'shock time' [ST], defined as MAP of less than 60 mm Hg), a second set of measurements was performed. The animals were then randomly assigned to receive a first-line therapy with the selective V_2_R-antagonist (1 μg/kg per hour; *n *= 7; Bachem Distribution Services AG, Weil am Rhein, Germany), AVP (0.05 μg/kg per hour, equivalent to 0.5 mU/kg per minute or 0.035 U/minute in a 70-kg patient; *n *= 7; American Regent Inc., Shirley, NY, USA), or normal saline (*n *= 7; B. Braun Melsungen AG, Melsungen, Germany). Open-label norepinephrine (Arterenol; Aventis Pharma, Frankfurt, Germany) was additionally titrated up to 1 μg/kg per minute to maintain MAP at 70 ± 5 mm Hg in all groups, if necessary. To ensure normovolemia, continuous infusions of balanced isotonic crystalloids (Sterofundin ISO; B. Braun Melsungen AG, Melsungen, Germany) and 6% hydroxyethyl starch 130/0.4 (Voluven; Fresenius Kabi, Bad Homburg, Germany) were infused at 8 and 4 mL/kg per hour, respectively, after ST. Additional fluids (crystalloid/colloid ratio of 2:1) were infused if hematocrit exceeded BL values during the 24-hour study period [14].

### Hemodynamic measurement, blood gas, laboratory, and histological analyses

Hemodynamic measurements, arterial and mixed venous blood gas, and laboratory analyses of variables of organ dysfunction and AVP plasma levels were performed at specific time points. Details on these measurements are provided in the supplemental digital content in Additional file 1.

### Immunohistochemical analyses

Following death, tissue samples were immediately stored for immunohistochemical analyses. Pulmonary concentrations of hemeoxygenase-1 (StressXpress Human HO-1 ELISA [enzyme-linked immunosorbent assay] Kit; Stressgen Bioreagents, Ann Arbor, MI, USA) and 3-nitrotyrosine (Hycult biotechnology 3-nitrotyrosine solid-phase ELISA; Cell Sciences, Canton, MA, USA) were determined as described previously [16,17].

### Statistical analyses

Sigma Stat 3.1 software (Systat Software, Inc., San Jose, CA, USA) was used for statistical analyses. Analysis-of-variance methodologies appropriate for two-factor experiments with repeated measures across time for each animal were used. Each variable was analyzed separately for differences among groups and differences across time and for group by time interaction. After confirmation of the significance of different group effects over time, *post hoc *pairwise comparisons among groups were performed using the Student-Newman-Keuls procedure to adjust for the elevated false-positive rate found otherwise in multiple testing. After 10 hours, no statistical analyses were performed, because the small number of animals alive in the placebo and the AVP group did not allow reliable testing anymore. Survival times were calculated using a log-rank test. Group differences were analyzed by pairwise multiple comparison with the Holm-Sidak test. Differences were considered statistically significant for *P *values of less than 0.05.

## Results

### Baseline characteristics

There were no differences among study groups in any of the investigated variables at BL and ST. Mean body weight (37 ± 1 kg) and time to onset of septic shock (7 ± 1 hours) did not differ between groups.

### Cardiopulmonary hemodynamics

**Figure 1:**
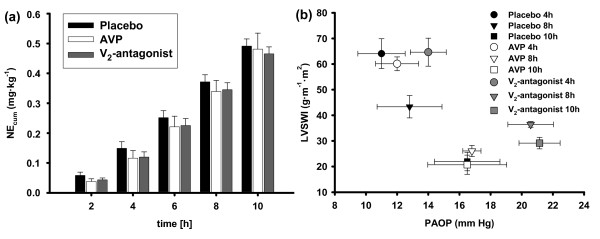
**Cumulative norepinephrine requirements (a) and left ventricular function curves (b)**. *n *= 7 each. AVP, arginine vasopressin; LVSWI, left ventricular stroke work index; NE_cum_, cumulative norepinephrine dose; PAOP, pulmonary artery occlusion pressure.

**Figure 2:**
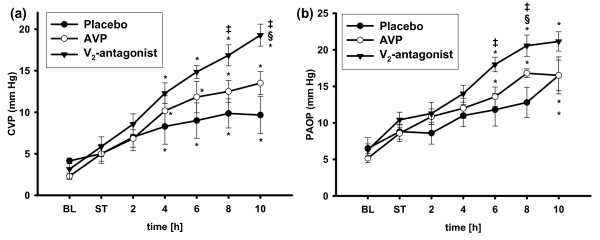
**Cardiac filling pressures**. Central venous pressure **(a) **and pulmonary artery occlusion pressure **(b)**. *P < 0.05 versus shock time (ST); ^‡^*P *< 0.05 versus placebo; ^§^*P *< 0.05 versus arginine vasopressin (AVP); *n *= 7 each. BL, baseline; CVP, central venous pressure; PAOP, pulmonary artery occlusion pressure.

Changes in cardiopulmonary variables are presented in Figures 1 and 2 and Table 1. Septic shock was characterized by decreases in MAP, systemic vascular resistance index, and left ventricular stroke work index (LVSWI) (ST: *P *< 0.001 versus BL each). All three treatment strategies maintained MAP within the target range of 70 ± 5 mm Hg for the first 4 hours after ST (4 hours: *P *< 0.01 versus ST each; Table 1). However, after the dose limitation for norepinephrine had been reached, MAP and systemic vascular resistance index fell significantly below ST values in all groups (10 hours: *P *< 0.05 versus ST each; Table 1). There were no statistically significant differences in cumulative norepinephrine requirements among study groups (Figure 1a).

**Table 1:** Cardiopulmonary variables and mesenteric blood flow

Variable	Group	Baseline	Shock time	4 hours	8 hours	10 hours
HR, beats per min	Placebo	96 ± 2	103 ± 4	123 ± 7^a^	115 ± 7	102 ± 5
	AVP	93 ± 2	101 ± 5	112 ± 6	99 ± 5^b^	100 ± 2
	V_2 _antagonist	95 ± 4	102 ± 3	112 ± 6^a^	115 ± 3^a,c^	101 ± 2
CI, L/min per m^2^	Placebo	5.5 ± 0.3	5.8 ± 0.5	8.6 ± 0.8^a^	7.9 ± 0.5^a^	5.8 ± 0.6
	AVP	5.2 ± 0.3	6.5 ± 0.4	8.5 ± 0.9	6.4 ± 0.8	5.4 ± 0.8
	V_2 _antagonist	5.3 ± 0.2	5.9 ± 0.3	9.7 ± 0.5^a^	8.2 ± 0.5^a^	7.1 ± 0.4
SVRI, dyne·s/cm^5 ^per m^2^	Placebo	1,285 ± 109	758 ± 52^d^	636 ± 60	463 ± 38^a^	457 ± 107^a^
	AVP	1,427 ± 101	664 ± 47^d^	596 ± 109	498 ± 84	479 ± 97^a^
	V_2 _antagonist	1,406 ± 25	714 ± 46^d^	509 ± 76^a^	388 ± 54^a^	464 ± 75^a^
MAP, mm Hg	Placebo	91 ± 4	58 ± 4^d^	66 ± 3^a^	55 ± 3	44 ± 3^a^
	AVP	93 ± 2	57 ± 1^d^	68 ± 2^a^	56 ± 4	43 ± 1^a^
	V_2 _antagonist	96 ± 2	58 ± 1^d^	68 ± 3^a^	54 ± 2	51 ± 3^a^
SVI, mL/m^2^	Placebo	59 ± 4	58 ± 7	78 ± 3^a^	68 ± 3	55 ± 7
	AVP	56 ± 3	64 ± 2	80 ± 7	63 ± 7	55 ± 9
	V_2 _antagonist	53 ± 2	57 ± 3	78 ± 7^a^	71 ± 5	70 ± 3
LVSWI, g/m per m^2^	Placebo	67 ± 3	41 ± 4^d^	64 ± 6^a^	43 ± 4	22 ± 4^a^
	AVP	67 ± 3	42 ± 2^d^	60 ± 3^a^	26 ± 2^b^	21 ± 4^a^
	V_2 _antagonist	65 ± 3	37 ± 2^d^	65 ± 5^a^	36 ± 1^c^	29 ± 2^c^
MPAP, mm Hg	Placebo	14 ± 1	20 ± 1^d^	22 ± 1	24 ± 2^a^	26 ± 2^a^
	AVP	15 ± 0	18 ± 1^d^	22 ± 1^a^	25 ± 1^a^	27 ± 2^a^
	V_2 _antagonist	15 ± 1	21 ± 1^d^	25 ± 2^a^	27 ± 1^a^	29 ± 1^a^
PVRI, dyne·s/cm^5 ^per m^2^	Placebo	106 ± 8	139 ± 22	119 ± 15	119 ± 12	144 ± 30
	AVP	124 ± 9	143 ± 8	90 ± 13^a^	81 ± 16^a^	150 ± 29
	V_2 _antagonist	129 ± 9	150 ± 9	121 ± 26	103 ± 8^a^	123 ± 10
Qma, % of baseline	Placebo	100 ± 0	109 ± 17	135 ± 27	94 ± 17	60 ± 10^a^
	AVP	100 ± 0	95 ± 7	118 ± 21	86 ± 16	41 ± 8^a^
	V_2 _antagonist	100 ± 0	95 ± 11	115 ± 11	75 ± 6	43 ± 8^a^

^a^*P *< 0.05 versus shock time; ^b^*P *< 0.05 versus placebo; ^c^*P *< 0.05 versus arginine vasopressin (AVP); ^d^*P *< 0.05 versus baseline; each group *n *= 7. CI, cardiac index; HR, heart rate; LVSWI, left ventricular stroke work index; MAP, mean arterial pressure; MPAP, mean pulmonary arterial pressure; PVRI, pulmonary vascular resistance index; Qma, mesenteric arterial blood flow; SVI, stroke volume index; SVRI, systemic.

LVSWI increased significantly in all groups at 2 and 4 hours (*P *< 0.05 versus ST each). Notably, LVSWI was higher in the V_2_R-antagonist group than in the AVP group at 8 and 10 hours (Table 1). Left ventricular contractility, expressed as a Starling-based relationship between LVSWI and preload (pulmonary artery occlusion pressure), was higher in animals treated with the V_2_R-antagonist than with placebo (Figure 1b). Cardiac index increased after ST. Heart rate was lower following AVP infusion than in both other groups (8 hours: *P *= 0.027 versus V_2_R-antagonist; *P *= 0.031 versus placebo; Table 1).

Central venous and pulmonary artery occlusion pressures, as surrogate variables of cardiac filling pressures, increased in all groups as compared with ST but were higher in animals treated with the V_2_R-antagonist as compared with both other groups (Figure 2a,b). Independently from the individual treatment regimen, mean pulmonary artery pressure increased during the study period (8 and 10 hours: *P *< 0.05 versus ST each; Table 1).

### Mesenteric blood flow

Mesenteric blood flow decreased in all groups (10 hours: *P *< 0.05 versus ST each; Table 1) without any statistically significant differences among groups.

### Pulmonary gas exchange and global oxygen transport

Besides a lower PaO_2_/FiO_2 _(arterial partial pressure of oxygen/fraction of inspired oxygen) ratio in the V_2_R-antagonist group compared with the placebo group at 4 hours (*P *= 0.039, Table 2), there were no statistically significant differences between study groups in variables of pulmonary gas exchange and global oxygen transport (Table 2).

**Table 2:** Hematocrit, electrolytes, acid-base balance, and global oxygen transport

Variable	Group	Baseline	Shock time	4 hours	8 hours	10 hours
Hct, %	Placebo	30 ± 2	28 ± 2	30 ± 2	30 ± 2	27 ± 2
	AVP	27 ± 2	26 ± 2	28 ± 2	27 ± 1	28 ± 2
	V_2 _antagonist	26 ± 1	25 ± 2	27 ± 2	26 ± 2	27 ± 1
Na^+^, mmol/L	Placebo	141 ± 1	140 ± 1	140 ± 1	140 ± 1	140 ± 1
	AVP	140 ± 1	139 ± 1	139 ± 1	139 ± 1	138 ± 1
	V_2 _antagonist	140 ± 0	139 ± 1	140 ± 1	140 ± 1	140 ± 1
K^+^, mmol/L	Placebo	4.1 ± 0.1	4.3 ± 0.2	4.4 ± 0.3	5.5 ± 0.3^a^	6.1 ± 0.3^a^
	AVP	3.8 ± 0.2	4.0 ± 0.2	4.1 ± 0.1	5.2 ± 0.3^a^	5.6 ± 0.4^a^
	V_2 _antagonist	3.9 ± 0.3	4.2 ± 0.3	4.3 ± 0.2	5.1 ± 0.3	5.5 ± 0.4^a^
Cl^-^, mmol/L	Placebo	108 ± 1	117 ± 2^b^	120 ± 1	124 ± 1^a^	125 ± 1^a^
	AVP	105 ± 1	113 ± 1^b^	118 ± 1	121 ± 1^a^	123 ± 1^a^
	V_2 _antagonist	108 ± 1	115 ± 2^b^	118 ± 2	121 ± 2	122 ± 2^a^
pH_a_, -log_10 _[H^+^]	Placebo	7.39 ± 0.01	7.30 ± 0.02^b^	7.20 ± 0.02	7.09 ± 0.04^a^	7.01 ± 0.06^a^
	AVP	7.42 ± 0.01	7.31 ± 0.02^b^	7.22 ± 0.02	7.05 ± 0.05^a^	7.04 ± 0.06^a^
	V_2 _antagonist	7.42 ± 0.02	7.33 ± 0.02^b^	7.28 ± 0.01	7.22 ±0.04^c,d^	7.11 ± 0.05^a^
PaO_2_/FiO_2_, mm Hg	Placebo	516 ± 23	458 ± 26	435 ± 43	217 ± 41^a^	149 ± 32^a^
	AVP	488 ± 23	492 ± 55	383 ± 27^a^	141 ± 25^a^	160 ± 19^a^
	V_2 _antagonist	465 ± 27	412 ± 26	313 ± 20^a,c^	153 ± 30^a^	140 ± 26^a^
SvO_2, _%	Placebo	78 ± 3	74 ± 4	80 ± 3	74 ± 1	60 ± 4^a^
	AVP	78 ± 1	76 ± 2	83 ± 4	70 ± 5	72 ± 4
	V_2 _antagonist	79 ± 2	78 ± 2	85 ± 2	78 ± 3	68 ± 4
DO_2_I, mL/min per m^2^	Placebo	731 ± 63	719 ± 83	1,105 ± 115^a^	918 ± 39	575 ± 92
	AVP	641 ± 58	739 ± 65	955 ± 128	749 ± 99	620 ± 85
	V_2 _antagonist	598 ± 36	664 ± 50	1,132 ± 139^a^	936 ± 50	707 ± 64
VO_2_I, mL/min per m^2^	Placebo	160 ± 12	179 ± 14	181 ± 19	172 ± 22	155 ± 21
	AVP	163 ± 13	167 ± 8	175 ± 8	144 ± 25	123 ± 18^a^
	V_2 _antagonist	128 ± 17	153 ± 10	163 ± 17	142 ± 13	132 ± 17
O_2_-ER, %	Placebo	23 ± 3	26 ± 3	18 ± 3	18 ± 2^a^	26 ± 2
	AVP	24 ± 2	25 ± 1	21 ± 6	21 ± 4	22 ± 4
	V_2 _antagonist	20 ± 1	23 ± 1	13 ± 1^a^	16 ± 2	20 ± 4

^a^*P *< 0.05 versus shock time; ^b^*P *< 0.05 versus baseline; ^c^*P *< 0.05 versus placebo; ^d^*P *< 0.05 versus arginine vasopressin; each group *n *= 7. AVP, arginine vasopressin; DO_2_I, oxygen delivery index; Hct, hematocrit; O_2_-ER, oxygen extraction rate; PaO_2_/FiO_2_, ratio of arterial partial pressure of oxygen and inspiratory oxygen fraction; pH_a_, arterial potentia hydrogenii; SvO_2_, mixed venous oxygen saturation, VO_2_I, oxygen consumption index.

### Capillary leakage

In all study groups, septic shock was characterized by a marked decrease in plasma protein concentrations (ST: *P *< 0.001 versus BL each) that progressed over the study period (8 hours: *P *< 0.001 versus ST each; Table 3). At the same time, there were no statistical differences in hematocrit within or among groups (Table 2), suggesting adequate fluid resuscitation. Cumulative positive net fluid balance was similar with all three treatment regimes (V_2_R-antagonist: 19 ± 1 mL/kg per hour; AVP: 17 ± 1 mL/kg per hour; placebo: 18 ± 2 mL/kg per hour).

**Table 3:** Surrogate parameters of organ (dys)function

Variable	Group	Baseline	Shock time	4 hours	8 hours
AST, U/L	Placebo	71 ± 7	76 ± 6	81 ± 14	112 ± 18^a^
	AVP	71 ± 7	78 ± 7	80 ± 12	77 ± 8
	V_2 _antagonist	72 ± 8	74 ± 8	58 ± 9	63 ± 10^b^
ALT, U/L	Placebo	7 ± 2	9 ± 1	9 ± 2	13 ± 3
	AVP	8 ± 3	11 ± 1	8 ± 2	11 ± 2
	V_2 _antagonist	8 ± 2	10 ± 3	5 ± 1	6 ± 1^b^
Bilirubin, mg/dL	Placebo	0.24 ± 0.02	0.24 ± 0.02	0.26 ± 0.04	0.25 ± 0.02
	AVP	0.25 ± 0.02	0.23 ± 0.02	0.23 ± 0.02	0.18 ± 0.04
	V_2 _antagonist	0.24 ± 0.02	0.23 ± 0.02	0.23 ± 0.03	0.16 ±0.03^b^
Plasma protein, mg/dL	Placebo	4.3 ± 0.2	1.9 ± 0.2^c^	1.2 ± 0.1^a^	0.7 ± 0.0^a^
	AVP	4.4 ± 0.2	2.1 ± 0.1^c^	1.2 ± 0.1^a^	0.9 ± 0.2^a^
	V_2 _antagonist	4.2 ± 0.3	1.9 ± 0.2^c^	1.2 ± 0.1	0.9 ± 0.2^a^
Creatinine, mg/dL	Placebo	0.8 ± 0.1	0.7 ± 0.1	1.1 ± 0.1	1.5 ± 0.1^a^
	AVP	0.7 ± 0.1	0.7 ± 0.1	0.7 ± 0.1	1.3 ± 0.2^a^
	V_2 _antagonist	0.8 ± 0.1	0.8 ± 0.1	0.8 ± 0.1	1.1 ± 0.2
Creatinine clearance, mL/min	Placebo	270 ± 82	228 ± 36	37 ± 10^a^	16 ± 3^a^
	AVP	254 ± 29	197 ± 42	214 ± 59^b^	24 ± 2^a^
	V_2 _antagonist	235 ± 43	198 ± 20	346 ± 52^b^	48 ± 15^a^

^a^*P *< 0.05 versus shock time; ^b^*P *< 0.05 versus placebo; ^c^*P *< 0.05 versus baseline; *n *= 7 each. ALT, alanine aminotransferase; AST, aspartate aminotransferase; AVP, arginine vasopressin.

### Metabolic changes and electrolytes

**Figure 3:**
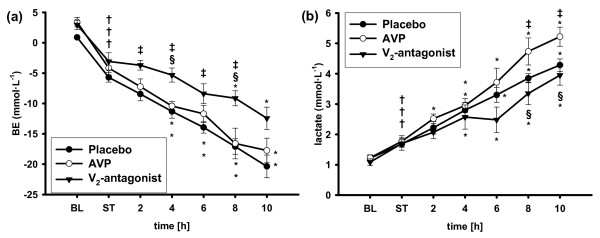
**Arterial base excess (a) and arterial lactate concentrations (b)**. ^†^*P *< 0.05 versus baseline (BL); *P < 0.05 versus shock time (ST); ^‡^*P *< 0.05 versus placebo; ^§^*P *< 0.05 versus arginine vasopressin (AVP); *n *= 7 each. BE, base excess.

Septic shock was associated with decreases in arterial pH and base excess (*P *< 0.05 versus BL each and *P *< 0.001 versus BL each, respectively) and increases in arterial lactate concentrations (*P *< 0.05 versus BL each) in all groups (Figure 3a,b and Table 2). These metabolic changes progressed during the observation period (8 hours: *P *< 0.001 versus ST each). However, the increase in arterial lactate concentration was attenuated (8 and 10 hours: *P *< 0.01 each), arterial base excess was less negative, and pH values were higher in the selective V_2_R-antagonist group as compared with the AVP and placebo groups after 8 hours (*P *< 0.05 each). Plasma concentrations of potassium and chloride increased in all groups during the study period (*P *< 0.05 versus ST each) without significant differences among groups.

### Laboratory variables of organ function and arginine vasopressin plasma levels

**Figure 4:**
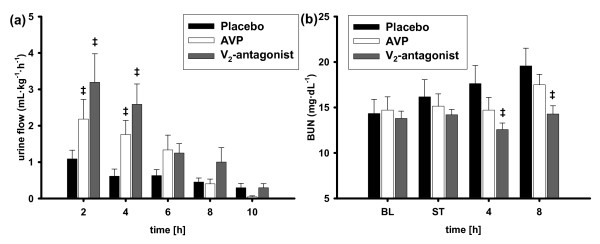
**Renal function**. ^‡^*P *< 0.05 versus placebo; *n *= 7 each. AVP, arginine vasopressin; BL, baseline; BUN, blood urea nitrogen; ST, shock time.

Alanine aminotransferase and aspartate aminotransferase activity as well as plasma concentrations of bilirubin were reduced by the selective V_2_R-antagonist as compared with placebo animals (8 hours: *P *< 0.05 each; Table 3). Renal dysfunction was evidenced by a progressive increase in blood urea nitrogen and plasma creatinine concentrations as well as a decrease in urine output and creatinine clearance in placebo animals (Figure 4 and Table 3). Infusion of the selective V_2_R-antagonist was associated with an increased creatinine clearance (4 hours: *P *< 0.001), a higher urine output (2 to 4 hours: *P *< 0.001 each), and lower blood urea nitrogen levels (4 to 8 hours: *P *= 0.031 and *P *= 0.023, respectively) as compared with the placebo group. There were no statistical differences in renal and liver function between the V_2_R-antagonist and the AVP group.

**Figure 5:**
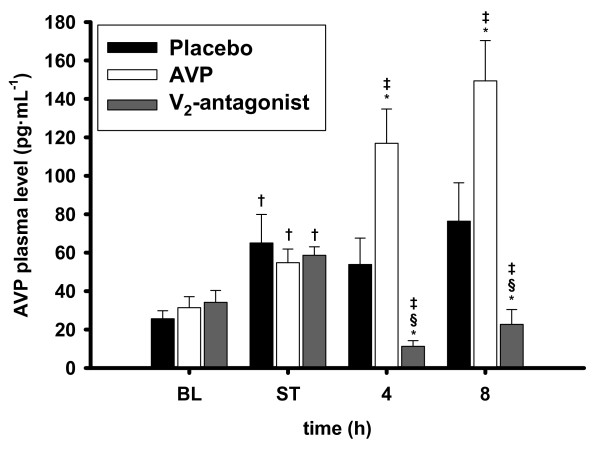
**Arginine vasopressin (AVP) plasma levels**. ^†^*P *< 0.05 versus baseline (BL); *P < 0.05 versus shock time (ST); ^‡^*P *< 0.05 versus placebo; ^§^*P *< 0.05 versus AVP; *n *= 7 each. BL, baseline.

The onset of septic shock was associated with an increase in AVP plasma levels as compared with BL in all groups (*P *< 0.05 versus BL each; Figure 5). Whereas AVP plasma levels remained constant in the placebo group, infusion of AVP increased AVP plasma levels up to 149 ± 21 pg/mL. Treatment with the selective V_2_R-antagonist led to a significant decrease of AVP plasma levels as compared with ST (*P *< 0.001) and with both other groups (4 to 8 hours: *P *< 0.05 versus placebo; *P *< 0.001 versus AVP).

### Immunohistochemical analyses

**Figure 6:**
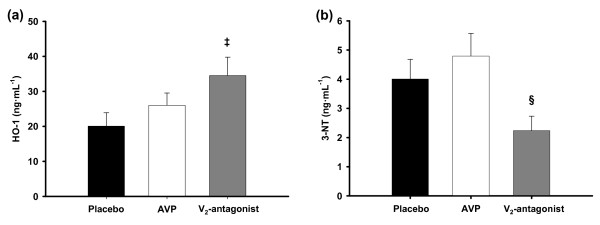
**Pulmonary hemoxygenase-1 (a) and 3-nitrotyrosine (b) concentrations**. ^‡^*P *< 0.05 versus placebo; ^§^*P *< 0.05 versus arginine vasopressin (AVP); *n *= 7 each. 3-NT, 3-nitrotyrosine; HO-1, hemeoxygenase-1.

Immunohistochemical analyses of lung tissue revealed an increase in hemeoxygenase-1 concentration in the selective V_2_R-antagonist group as compared with placebo animals (*P *= 0.047; Figure 6a). In addition, pulmonary 3-nitrotyrosine concentrations were lower in animals treated with the selective V_2_R-antagonist as compared with AVP (*P *= 0.017; *P *= 0.056 versus placebo; Figure 6b).

### Survival time

**Figure 7:**
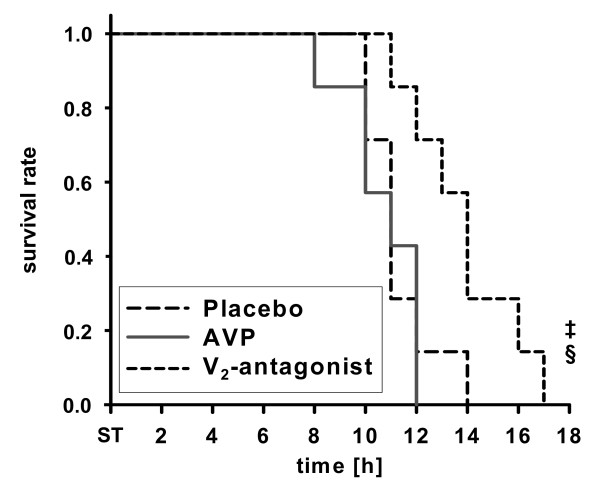
**Kaplan-Meier survival curve**. ^‡^*P *< 0.05 versus placebo; ^§^*P *< 0.05 versus arginine vasopressin (AVP); *n *= 7 each. ST, shock time.

All animals died within 17 hours after the onset of septic shock (Figure 7). Sheep treated with the selective V_2_R-antagonist had a longer survival time (14 ± 1 hours) than animals that received AVP (11 ± 1 hours; *P *= 0.007) or placebo (11 ± 1 hours; *P *= 0.025). There were no significant differences in survival time between the AVP and sole norepinephrine groups (*P *= 0.727).

## Discussion

The major findings of the present study are that first-line therapy with the selective V_2_R-antagonist (a) stabilized cardiopulmonary hemodynamics as effectively, (b) increased cardiac filling pressures, (c) attenuated metabolic acidosis, (d) limited myocardial and renal dysfunction, (e) reduced AVP plasma levels, (f) attenuated tissue injury secondary to nitrosative stress, and (g) slightly prolonged survival in early volume-resuscitated, hyperdynamic ovine septic shock when compared with placebo and AVP infusion.

The relative vasopressin deficiency [18] represents the rationale for the use of AVP in the treatment of septic shock. However, only one third of septic shock patients suffer from low AVP plasma levels [19]. Typically, endogenous AVP secretion increases in the early phase of septic shock and decreases thereafter. Since V_2_Rs are involved in several characteristic pathways of septic shock [4-11,20], selective V_2_R-antagonism rather than V_2_R-stimulation (for example, via AVP infusion) may be advantageous under these circumstances.

In the present study, AVP plasma levels increased with the onset of septic shock in all groups and remained at this level in the placebo group during the whole study period. The absence of a 'relative vasopressin deficiency' may be one reason for the ineffectiveness of AVP in reducing norepinephrine requirements as compared with standard treatment with norepinephrine in the placebo group. Another potential explanation is that the AVP dose of 0.05 μg/kg per hour (equivalent to 0.5 mU/kg per minute or 0.035 U/minute in a 70-kg patient) might have been insufficient for the fulminant injury in our model (100% mortality within 17 hours). The latter assumption is in harmony with the observation made in VASST that AVP reduced mortality in less severe septic shock but not in the more severe septic shock population [2]. In this context, Torgersen and colleagues [21] recently reported that, in patients with sepsis-induced vasodilatory shock, a supplementary infusion of 0.067 U/minute AVP was more effective in restoring MAP and reducing norepinephrine requirements than the recommended low dose of 0.033 U/minute.

Interestingly, infusion of the selective V_2_R-antagonist reduced AVP plasma levels as compared with AVP- and placebo-treated animals. This finding appears to be surprising at first glance. In this context, however, it may be of importance that AVP has a positive feedback on its own release via V_2_R [22]. Therefore, it is most likely that inhibition of this mechanism has accounted for the low AVP plasma levels noticed in the V_2_R-antagonist group.

Another interesting result of the present study is that the selective V_2_R-antagonist was as effective as AVP in stabilizing cardiopulmonary hemodynamics without increasing volume and norepinephrine requirements. The reduction in metabolic acidosis by the V_2_R-antagonist - as suggested by higher pH values, less negative base excess, and lower lactate levels as compared with both other groups - probably reduced systemic vasodilation [23] and contributed to an improved efficacy of norepinephrine by increasing the adrenergic receptor sensitivity [24,25].

In this context, it may also be important that extrarenal V_2_R mediates vasorelaxant effects [4], thereby decreasing MAP and vascular resistance not only in the experimental setting [26] but also in humans [6,27].

In addition, the increased cardiac filling pressures in animals treated with the V_2_R-antagonist may have improved systemic hemodynamics. This assumption is supported by the Starling-based relationship between LVSWI and preload (Figure 1b). Since hematocrit remained stable in all groups, the increased preload in the V_2_R-antagonist group has most likely been caused by a mobilization of fluid from venous capacity vessels.

Whereas both the V_2_R-antagonist and AVP increased urine output and creatinine clearance as compared with placebo animals, the V_2_R-antagonist additionally reduced blood urea nitrogen versus placebo. A protective effect of V_2_R-antagonism on renal function is supported by Rondaij and colleagues [28], who reported that V_2_R agonism caused histological renal lesions in rats and that these lesions were prevented by V_2_R-antagonism.

In addition, the reduction of oxidative stress, as suggested by immunohistochemical analyses of lung tissues, probably contributed to the attenuated organ dysfunction in the V_2_R-antagonist group as compared with placebo and AVP. Whereas 3-nitrotyrosine represents a stable *in vivo *biomarker of the highly cytotoxic compound peroxynitrite [29], hemeoxygenase-1 has been reported to provide cytoprotective effects [30].

Attenuation of cardiovascular, metabolic, and renal function as well as nitrosative stress in response to first-line V_2_R-antagonist infusion led to a slight prolongation in survival time as compared with AVP and placebo treatment. Such effects on survival time were not observed with AVP, suggesting that its V_2_R agonism might potentially be disadvantageous.

This study has some limitations that we want to acknowledge. In the absence of source control and antibiotic therapy, the present model was associated with a high mortality (all animals died within the observation period). As a consequence, effects of the investigated therapeutic approaches could be analyzed only during the acute phase of the injury. In addition, the present study was not designed primarily for detecting differences in mortality. For these reasons, data on survival times in the current study should not be overestimated. In addition, conclusions on the clinical relevance of the present findings are limited by the experimental design and the use of previously healthy animals, whereas the majority of patients typically suffer from comorbidities. Finally, the risk of false-positive results in a study with numerous outcome variables and time points has to be taken into consideration.

## Conclusions

To our knowledge, this is the first study providing evidence that, under conditions with high endogenous AVP plasma levels, first-line treatment with the selective V_2_R-antagonist supplemented with open-label norepinephrine improves cardiovascular, metabolic, liver, and renal function and slightly prolongs survival when compared with first-line therapy with AVP or placebo in ovine septic shock. On the basis of the present findings, the use of selective V_2_R-antagonists potentially represents a new therapeutic approach in the early stage of septic shock.

## Key messages

• V_2_-receptor stimulation aggravates sepsis-induced vasodilation, fluid accumulation, and microvascular thrombosis.

• Arginine vasopressin (AVP) infusion in septic shock may be less effective when endogenous AVP plasma levels are high.

• In ovine septic shock, selective V_2_-receptor-antagonism supplemented with open-label norepinephrine stabilized cardiovascular hemodynamics as effectively as combined AVP and open-label norepinephrine.

• Selective V_2_-receptor-antagonism attenuated metabolic, liver, and renal dysfunction as compared with AVP and placebo therapy in ovine septic shock.

• Selective V_2_-receptor-antagonism might represent a useful therapeutic option in septic shock under conditions with high endogenous AVP plasma levels.

## Abbreviations

AVP: arginine vasopressin; BL: baseline; ELISA: enzyme-linked immunosorbent assay; LVSWI: left ventricular stroke work index; MAP: mean arterial pressure; ST: shock time; V_1a_R/V_2_R: V_1a_/V_2 _receptor; VASST: Vasopressin and Septic Shock Trial.

## Competing interests

The authors declare that they have no competing interests.

## Authors' contributions

SR designed and performed the experiment, summarized and analyzed the data, and wrote the manuscript. CE designed and performed the experiment, summarized and analyzed the data, and edited the manuscript. MW and AM designed the experiment, analyzed the data, and edited the manuscript. ML, EW, MD, HVA, and DLT analyzed the data and edited the manuscript. A-KS, EL, and TGK performed the experiment and summarized the data. All authors read and approved the final manuscript.

## Supplementary Material

Additional file 1 **Supplemental Digital Content**. Additional information on the methods and procedures applied in the present study [31-33]. Click here for file
