# Supplementary material for: Role of selective V2-receptor-antagonism in septic shock: a randomized, controlled, experimental study
Source: Crit Care. 2010 Nov 5;14(6):R200. doi: 10.1186/cc9320 (PMC3220000; doi:10.1186/cc9320)
Supplement: Additional file 1 — Supplemental Digital Content. Additional information on the methods and procedures applied in the present study [31-33]. [file cc9320-S1.DOC]

**Role of selective V_2_-receptor antagonism in septic shock:**

**a randomized, controlled, experimental study**

**Supplemental Digital Content**

Sebastian Rehberg, MD^1^; Christian Ertmer, MD^1^; Matthias Lange, MD, PhD^1^; Andrea Morelli, MD^2^; Elbert Whorton, PhD^3^; Martin Dünser, MD^4^; Anne-Katrin Strohhäcker, MS^1^; Erik Lipke, MS^1^; Tim G. Kampmeier, MD^1^; Hugo Van Aken, MD, PhD, FRCA, FANZCA^1^; Daniel L. Traber, PhD^5^; Martin Westphal, MD, PhD^1^

^1^Department of Anesthesiology and Intensive Care, University of Muenster, Muenster, Germany; ^2^Department of Anesthesiology and Intensive Care, University of Rome, “La Sapienza”, Rome, Italy; ^3^Department of Biostatistics and Epidemiology, University of Texas Medical Branch, Galveston, Texas, USA; ^4^Department of Intensive Care Medicine, Inselspital, Medical University of Bern, Bern, Switzerland; ^5^Investigational Intensive Care Unit, Department of Anesthesiology, University of Texas Medical Branch, Galveston, Texas, USA

**Methods**

*Instrumentation*

Sheep were fasted for 24 h before the experiment began, but had free access to water [14]. After induction of anesthesia with i.m. injection of S-ketamine (Ketanest S®; 10 mg∙kg^-1^; Parke-Davis Berlin, Freiburg, Germany) and midazolam (Dormicum®; 0.3 mg·kg^-1^; Hoffmann-La Roche, Ltd., Grenzach-Wyhlen, Germany), the animals were weighed and placed on the operating table in supine position. After canulation of the left jugular vein (18 G catheter, BD Insyte-W, Vialon™ Material, Madrid, Spain), anesthesia was maintained using a continuous infusion of R/S-ketamine 10% (6 mg∙kg^-1^∙h^-1^, Ceva Tiergesundheit GmbH, Düsseldorf, Germany) and midazolam (0.3 mg∙kg^-1^∙h^-1^). Following bolus administration of 6 mg∙kg^-1^ R/S-ketamine and 0.3 mg∙kg^-1^ midazolam, the trachea was intubated (Tracheal tube 9.0, Rüsch GmbH, Kernen, Germany). Mechanical ventilation was performed using a volume-controlled mode (Dräger-AV1, Drägerwerke AG, Lübeck, Germany) with an initial inspired oxygen fraction of 0.3, a positive endexpiratory pressure of 5 cmH_2_O, an inspiratory/expiratory time ratio of 1:2 and a tidal volume of 9 mL∙kg^-1^ [31]. Notably, the dead space of the airways and the pulmonary compliance in sheep is larger than in humans. This information needs to be considered, when comparing the applied tidal volume with the recommendation of 6 mL∙kg^-1^ of the Acute Respiratory Distress Syndrome Network [32]. Respiratory variables were adjusted to ensure normoxemia (arterial partial pressure of oxygen: 80 - 120 torr (mmHg)) and normocapnia (arterial partial pressure of carbon dioxid: 35 - 45 torr (mmHg)), as measured by serial blood gas analyses, as long as possible. Anesthesia was additionally supplemented by inhalative isoflurane (1-1.5 vol% endtidal; Abbott GmbH, Wiesbaden, Germany) and nitrous oxide.

A left femoral arterial catheter (18-gauge Leader Cath; Vygon, Aachen, Germany) and an indwelling pulmonary artery catheter (7.5-Fr Edwards Swan Ganz; Edwards Critical Care Division, Irvine, CA) were then placed in all animals. The pulmonary artery catheter was inserted through an introducer sheath (8.5-Fr catheter introducer set; pvb Medizintechnik GmbH, Kirchseeon, Germany) via the right jugular vein. In addition, a balloon catheter was introduced into the urinary bladder (Porgès S.A., Le Plessis-Robinson Cedex, France) to monitor urinary output.

*Surgical Procedures*

Continuous i.v. infusion of crystalloids (4 mL∙kg^-1^∙h^-1^; Sterofundin ISO®, B. Braun, Melsungen, Germany) was started to prevent perioperative dehydration. Blood loss was replaced by equal volumes of 6% HES 130/0.4 (Voluven®, Fresenius Kabi, Bad Homburg, Germany), if necessary. A midline laparotomy was performed and an ultrasonic flow-probe (T208 Transonic volume flow meter, Transonic System Inc., New York, NY) was positioned around the superior mesenteric artery. The cecum was incised and feces (1.5 g∙kg^-1^ of body weight) were collected in a 100 mL syringe under sterile conditions to avoid fecal contamination before carrying out baseline measurements. Finally, the cecum and the abdominal cavity were closed by fascial and cutaneous sutures. A 25-F suction catheter remained *in situ* to allow injection of autologous feces into the peritoneal cavity at a later time point. After the surgical preparation, animals were turned to the prone position and allowed to recover for 1 h. Two sheep died during the induction of septic shock prior to randomization due to pulmonary hypertension and consecutive right heart failure and were excluded from the study. The entire experiment was carried out in 21 animals.

*Hemodynamic Monitoring*

Following instrumentation, intravascular catheters were connected to calibrated pressure transducers (DTX pressure transducer; Ohmeda KG, Erlangen, Germany) and a physiologic recorder (Hellige Servomed; Hellige, Freiburg, Germany) to monitor heart rate, mean arterial pressure, mean pulmonary arterial pressure, central venous pressure, and pulmonary arterial occlusion pressure. Core body temperature was continuously measured by the thermistor positioned at the tip of the pulmonary artery catheter. The thermodilution technique was applied to measure cardiac output with an average of three 10 mL injections of cold (2-5°C) isotonic saline solution as indicator (9520A cardiac output computer; Edward Lifescience, Irvine, CA). Cardiac index, systemic and pulmonary vascular resistance index, stroke volume as well as left ventricular stroke work indices were calculated using standard equations [33].

*Measurements*

Hemodynamic measurements, arterial and mixed venous blood gas samples were analyzed at baseline, shock time (defined as mean arterial pressure <60 mmHg), and every hour after the onset of septic shock. At baseline, shock time, 4 h and 8 h after shock time, arterial blood was withdrawn (9 mL ethylene diamine-tetracetate [EDTA] and 7.5 mL lithium-heparin), immediately centrifuged for 10 min at 3000 rpm, and the isolated plasma stored at -70°C for the determination of variables of organ function at a later time point. Similarly, urine samples (3 mL each) were taken for laboratory analyses of creatinine concentrations.

To determine hematocrit, arterial lactate concentrations, arterial and mixed-venous oxygen saturations, blood gas samples were analyzed with an ABL 625 blood gas analyzer (Radiometer Copenhagen, Copenhagen, Denmark). In addition, arterial partial pressures of oxygen and carbon dioxide as well as pH were assessed. The arterial base excess was calculated from pH and arterial partial pressure of carbon dioxide. Oxygen delivery index, oxygen consumption index and oxygen extraction rate were determined using standard formulae [33].

*Laboratory Variables*

Laboratory analyses were performed to determine alanine aminotransferase, aspartate aminotransferase activity and plasma concentrations of total proteins, bilirubin, creatinine and blood urea nitrogen as well as urine levels of creatinine (Hitachi 747 Automatic Analyzer; Böhringer, Mannheim, Germany). Creatinine clearance was calculated using a standard equation [33]. Plasma levels of AVP were determined using a radioimmunoassay (Vasopressin RIA; Bühlmann, Allschwill, Switzerland) as previously described [14].
